# Kinetics reshape antitumor immunity: Timing, duration, and combination are of importance for successful cancer immunotherapy

**DOI:** 10.1002/ctm2.1420

**Published:** 2023-09-18

**Authors:** Seung Mo Jin, Yeon Jeong Yoo, Yong Taik Lim

**Affiliations:** ^1^ Department of Nano Science and Technology, Department of NanoEngineering, SKKU Advanced Institute of Nanotechnology (SAINT), School of Chemical Engineering, and Biomedical Institute for Convergence at SKKU Sungkyunkwan University Suwon Republic of Korea

## 1 THE LIMITATION OF CURRENT CANCER IMMUNOTHERAPY

The current immune checkpoint blockade therapy (ICBT) still has a low response rate, which ranges from approximately 5%–30% depending on the target tumour.^1^ The low response rate is related to the presence of few T cells (“cold tumours”) and the exhaustion of relevant immune cells, such as dendritic cells (DCs) and T cells, in the immunosuppressive tumour microenvironment (TME). Although various immune modulatory components such as Toll‐like receptor (TLR) agonists have been used for the activation of resting antigen‐presenting cells (APCs) and the generation of antigen‐specific T cells, they ultimately induce exhausted APCs and T cells, resulting in suboptimal cancer immunotherapy.^2^, ^3^, ^4^ Therefore, the strategy of tailoring innate immunity is particularly important for achieving effective cancer immunotherapy. In particular, novel immune modulation strategies to achieve robust innate and adaptive immune responses without inducing exhausted immune cells should be developed. To address the issue, the fact that the maturation of APCs is regulated not only by the nature of the APCs maturation stimuli but also by the duration, combination, and timing of the stimulations, which collectively affect subsequent T cell responses, should be considered in the design of immunomodulatory components for the APCs activation.

## 2 KINETIC IMMUNE MODULATION: TIMING, DURATION, AND COMBINATION

### 2.1 Kinetic immune responses by t‐TLR7/8a

**FIGURE 1:**
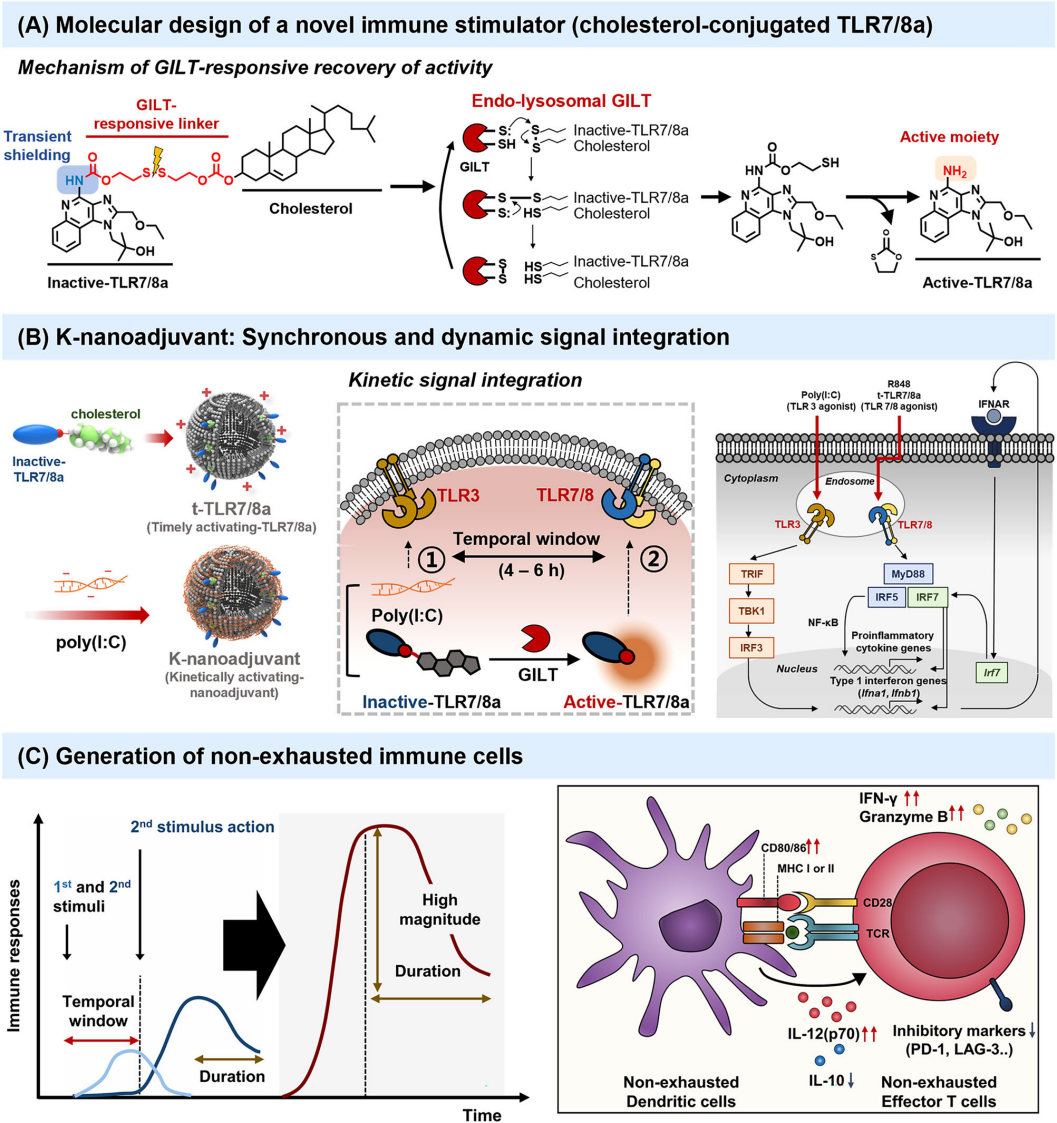
**Kinetically activating nanoadjuvant (K‐nanoadjuvant) that dynamically coordinates innate immune stimuli activation**. (A) Molecular design of gamma‐interferon‐inducible lysosomal thiol reductase (GILT)‐responsive time‐dependent activating immunomodulator, cholesterol‐conjugated TLR7/8a. (B) K‐nanoadjuvant for synchronous and dynamic integration of innate immune stimuli. (C) Generation of non‐exhausted immune cells through dynamic signal integration of K‐nanoadjuvant. Adapted.^5^ Copyright 2023 Springer Nature.

To realize the kinetic immune modulation through a single cell level, we developed timely activating TLR7/8a (t‐TLR7/8a), which activates as an immune stimulant in a time‐dependent manner.^5^ The design of t‐TLR7/8a was based on transient chemical shielding of the putative active site in TLR7/8a, specifically the C4 amine, which is known to interact with TLR7/8 through hydrogen bonding and recovery of activity via a chemical linker that can be cleaved in response to a specific signal within the endo/lysosomal microenvironment, such as gamma‐interferon‐inducible lysosomal thiol reductase (GILT)^6^ (Figure 1A). Our study demonstrates that GILT, which is the only enzyme known to catalyze disulfide bond reduction in the endocytic pathway with an optimal pH of 4.5–5.5, facilitates the reduction of the disulfide bond of dormant TLR7/8a and the corresponding recovery of the active site of TLR7/8a in endo/lysosomes.^5^, ^7^, ^8^ Notably, to the best of our knowledge, this study is the first that utilizes GILT as a trigger for the dynamic recovery of transiently dormant immunostimulants, providing a novel approach for dynamic immune modulation of APCs. Compared to conventional TLR7/8a (R848), t‐TLR7/8a demonstrated enhanced effectiveness in inducing sustained interleukin (IL)‐12p70 from bone‐marrow‐derived cells (BMDCs).^5^ In BMDCs and T cell co‐culture system, durable IL‐12p70 secretion by t‐TLR7/‐stimulated dendritic cells, promotes the differentiation of T helper 1 (Th1) cell of CD4^+^ T cells and mediates effective adaptive immune responses by reducing the PD‐1 expression on exhausted CD8^+^ T cells.^5^

### 2.2 Combinatorial codes of K‐nanoadjuvant

Through the systemic in vitro analysis, we observed that a specific time window between certain combined stimulants can maximize the synergistic effect. Especially, the synergistic effect of dual TLR agonists on IL‐12p70 production was maximized when R848 (TLR7/8a) was administered after Lipopolysaccharides (TLR4a) or poly(I:C) (TLR3a) treatment of BMDCs within a 4–8 h ‘time window’.^5^ This time window appeared attractive for the induction of strong DC and T cell responses by vaccination. The hidden mechanism for the optimized combinatorial code was disclosed through the type I interferon axis knockout mice (IFNAR1 KO) or TBK inhibitor treatment assay (Figure 1B).^5^ It was shown that IRF3‐dependent synthesis of type I interferon through TLR3 or TLR4 activation further acts in an autocrine and/or paracrine feedback loop to induce IRF7 synthesis. Only after being induced, it amplifies TLR7 or TLR8 signals by promoting sustained and enhanced type I interferon production and IL‐12p70 synthesis.

Inspired by the combinatorial code of innate immune stimuli, we developed a novel kinetically activating nanoadjuvant (K‐nanoadjuvant), entailing the co‐incorporation of t‐TLR7/8a and poly(I:C) within a nanoliposome formulation (Figure 1B).^5^ This approach enables the simultaneous delivery of t‐TLR7/8a and TLR3a into APCs within a single nanoliposome, thereby orchestrating an optimal order, duration, and time window for TLR activation in a synchronous manner.

### 2.3 Generation of non‐exhausted immune cells by K‐nanoadjuvant

We investigated the capacity of K‐nanoadjuvant to modulate immune responses in both the tumour‐draining lymph nodes (TDLNs) and TME, favouring T‐cell‐based antitumor immunity in B16OVA melanoma and TC‐1 tumour models. Compared with a soluble admixture of R848 and poly(I:C), mice immunized with K‐nanoadjuvant exhibited a substantial increase in antigen‐specific CD8^+^ T cells with high TME infiltration, and surprisingly, without increasing the expression of exhaustion markers (PD‐1, TIM‐3, and LAG‐3) (Figure 1C).^5^ Notably, neutralizing IL‐12 with anti‐IL‐12 significantly abrogated the effects of K‐nanoadjuvant on T cells, natural killer (NK) cells, and NKT cells activation within both the TDLNs and the TME and decreased the antitumor efficacy of K‐nanoadjuvant.^5^ Crucially, IL‐12 blockade led to elevated PD‐1 expression in CD8^+^ T cells in the TME, underlining the pivotal role of up‐regulated IL‐12 secretion by K‐nanoadjuvant in promoting the generation of non‐exhausted CD8^+^ T cells.^5^ This underscores IL‐12 as the key mediator linking K‐nanoadjuvant‐mediated innate immune stimulation and subsequent adaptive immune responses.

## 3 PROSPECTS: BEYOND CANCER IMMUNOTHERAPY

We have implemented a dynamic immune modulation concept wherein dual TLR agonists are designed to act timely within a ‘temporal window’, thereby enhancing the anticancer effect while circumventing immune exhaustion. There have been no previous studies on the dynamic immune modulation of APCs using well‐designed immune stimulants, and the effects of such dynamic immune modulation on both innate and adaptive immune responses remain unprecedented before our results. Moreover, the strategy of ‘dormancy (benign) at off‐target sites and recovery (active) at the target site’ through K‐nanoadjuvant proposed in this study can be extended as an approach to minimize undesirable immune‐related side effects of various TLR agonists at off‐target sites, while maximizing the therapeutic efficacy at intended sites.

In terms of the potential for clinical translation, confirmation of GILT presence in human moDCs suggests that the immunomodulatory properties of K‐nanoadjuvant can indeed be extended to humans. Considering that various commercial adjuvants have been combined with multiple immune stimulants, K‐nanoadjuvant, which optimized the combinatorial code of TLR7/8a with TLR3a for optimal signal integration with nanoliposome formulation, could be readily translated into the clinical application following preclinical studies. Furthermore, by orchestrating t‐TLR7/8a with various innate immune stimulants that have been clinically studied, this innovative strategy possesses the potential to broaden the spectrum of treatment applications, encompassing respiratory infectious diseases like severe acute respiratory syndrome coronavirus 2 as well as diverse forms of cancer.

## CONFLICT OF INTEREST STATEMENT

The authors declare no conflict of interest.
